# Building the Case for Interdisciplinary Care, One Patient at a Time

**DOI:** 10.1097/PG9.0000000000000148

**Published:** 2021-12-16

**Authors:** Miguel Saps

**Affiliations:** From the Division of Pediatric Gastroenterology, Hepatology and Nutrition, University of Miami, Mailman Center, Miami, FL.

Hypermobile Ehlers-Danlos syndrome (hEDS), Postural Orthostatic Tachycardia Syndrome (POTS), and mast cell activation syndrome are rare diseases. The prevalence of EDS at the community level is approximately two in 10,000 (1), while POTS is diagnosed in approximately one in 10,000 (2), individuals. The prevalence of mast cell activation syndrome is unknown but is thought to be infrequent (3). Despite being rare, patients with a combination of these diagnoses frequently consult at specialized clinics. The complexity of these patients and the severity of their comorbidities can be overwhelming to families and clinicians alike. Severe, unremitting and disabling abdominal pain and feeding problems prompt patients to seek gastroenterology consultations.

Evidence-based education is key to establishing a trustful relationship with children and families. This is particularly important for patients with multiple diagnoses. Physicians often struggle to explain the association between POTS, hEDS, and mast cell activation syndrome. There is a dearth of data explaining their relationship. A systematic review of the literature found no studies that could satisfactorily explain the pathophysiological link between these entities (3). They concluded that studies that proposed the existence of a true relationship were either based on outdated diagnostic criteria or simply biased. A recent editorial did not agree with this premise (4); thus, the basis of this relationship remains controversial.

Community-based studies can add some clarity to the “real” frequency of association of disorders. School-based studies found that functional gastrointestinal disorders (FGIDs) and isolated joint hypermobility—which is frequently wrongly confused for hEDS—are common (5). However, despite that, many children with joint hypermobility in the study met diagnosis for FGIDs; FGIDs were equally present among children with and without joint hypermobility. In contrast, POTS was rare among schoolchildren, yet again, equally present in children with and without FGIDs (5). Among more than 300 children examined for the study, only one child had both, joint hypermobility and POTS and no children had a combination of POTS, functional abdominal pain and joint hypermobility.

Considering the low prevalence of the association of these disorders at the community level, one has to wonder what can explain the seemingly high rate of consultations for a combination of connective tissue, autonomic, and functional abdominal pain disorders. In view of the paucity of data on the frequency of consultations to pediatric gastroenterology for this combination of disorders, we cannot exclude the possibility of recall bias. We frequently remember the most complicated patients that required extensive medical attention more vividly. Alternatively, patients with such a combination of disorders may in fact, have a higher representation among GI consultations compared with their prevalence in the community. It would be logical that the sickest patients consult the most. However, it is not unusual to see patients in clinic that were mislabeled with these diagnoses by parents without medical knowledge. Currently, medical information is more likely to be disseminated through internet sites rather than medical journals. Families are frequently unprepared to understand the information published or to distinguish between trustworthy sites and those of less repute that propose unscientific diagnosis, testing, and treatments. Confronted with families looking for unusually extensive diagnostic testing, physicians have two options, repeat or send new tests in search of another elusive uncommon organic diagnosis, or focus the consultation on providing reassurance and education on the biopsychosocial aspects of the patient’s symptoms.

In the current issue of JPGN-Reports, Kilgore et al published a case report that exemplifies how patients can benefit from the adoption of interdisciplinary care and a biopsychosocial approach (6). A key aspect to the care of complex GI patients is coordination of care by an interdisciplinary team with a unified message that is reinforced by all members of the team. The patient in this report not only received interdisciplinary and specialized hospital care but also benefitted from continuation of care in the outpatient setting. The patient was managed by an interdisciplinary team that included pediatric gastroenterology, chronic pain, complex care pediatrician, social work, and patient representative. The plan included pelvic floor physical therapy, feeding therapy, dietician appointments, cognitive behavioral therapy, and monthly visits to specialized Ehlers-Danlos and neurogastroenterology/motility clinics. Unfortunately, Kilgore’s et al’s approach to the evaluation and management of complex patients cannot be easily reproduced. Few hospitals recognize the importance of demonstrating readiness to invest the considerable resources needed to provide excellence of care to complex GI patients. Hospital administrators and the public in general should be aware of the importance of having these resources available in each community. It is time to take advantage of the opportunities provided by the internet to educate parents on the benefits of the interdisciplinary care and to reinforce the need of creating centers of excellence to care for complex patients.
